# Selecting renal replacement therapies: what do African American and non-African American patients and their families think others should know? A mixed methods study

**DOI:** 10.1186/1471-2369-14-9

**Published:** 2013-01-14

**Authors:** Nicole DePasquale, Patti L Ephraim, Jessica Ameling, Lapricia Lewis-Boyér, Deidra C Crews, Raquel C Greer, Hamid Rabb, Neil R Powe, Bernard G Jaar, Luis Gimenez, Priscilla Auguste, Mollie Jenckes, L Ebony Boulware

**Affiliations:** 1 Welch Center for Prevention, Epidemiology and Clinical Research, Johns Hopkins Medical Institutions, Baltimore, MD, 21205, USA; 2 Division of General Internal Medicine, Johns Hopkins University School of Medicine, Baltimore, MD, 21205, USA; 3 Department of Epidemiology, Johns Hopkins Bloomberg School of Public Health, Baltimore, MD, 21205, USA; 4 Division of Nephrology, Johns Hopkins Medical Institutions, 2024 E. Monument Street, Suite 2-600, Baltimore, MD, 21205, USA; 5 University of California San Francisco, San Francisco, CA, 94110, USA; 6 San Francisco General Hospital, San Francisco, CA, 94110, USA; 7 Good Samaritan Hospital of Maryland, Baltimore, MD, 21239, USA; 8 Nephrology Center of Maryland, Baltimore, MD, 21239, USA

**Keywords:** Decision-making, Renal replacement therapy, Family members, African American

## Abstract

**Background:**

Little is known regarding the types of information African American and non-African American patients with chronic kidney disease (CKD) and their families need to inform renal replacement therapy (RRT) decisions.

**Methods:**

In 20 structured group interviews, we elicited views of African American and non-African American patients with CKD and their families about factors that should be addressed in educational materials informing patients’ RRT selection decisions. We asked participants to select factors from a list and obtained their open-ended feedback.

**Results:**

Ten groups of patients (5 African American, 5 non-African American; total 68 individuals) and ten groups of family members (5 African American, 5 non-African American; total 62 individuals) participated. Patients and families had a range (none to extensive) of experiences with various RRTs. Patients identified morbidity or mortality, autonomy, treatment delivery, and symptoms as important factors to address. Family members identified similar factors but also cited the effects of RRT decisions on patients’ psychological well-being and finances. Views of African American and non-African American participants were largely similar.

**Conclusions:**

Educational resources addressing the influence of RRT selection on patients’ morbidity and mortality, autonomy, treatment delivery, and symptoms could help patients and their families select RRT options closely aligned with their values. Including information about the influence of RRT selection on patients’ personal relationships and finances could enhance resources’ cultural relevance for African Americans.

## Background

Patients’ decisions to select an initial renal replacement therapy (RRT) for end-stage renal disease (ESRD) are complex. The various forms of dialysis and transplantation differ not only in their impacts on patients’ future survival and quality of life [1-4], but also in important characteristics which could influence patients’ preferences, including treatment invasiveness [3,5], length of time for treatment delivery [5], and treatment requirements for self-care or family involvement [3]. Despite this, evidence suggests patients are often not fully informed about the availability of various RRT options or how these options differ from one another, even when initiated under the routine care of nephrologists [6-8]. Awareness of the availability and features of RRT options may be particularly poor among African Americans, who have significantly lower rates of initiating self-care treatment modalities such as live kidney transplantation (LKT) when compared to their non-African American counterparts [8,9].

Patients [10,11], nephrology professionals [12,13], and policy makers [14] have recently begun to emphasize the importance of informing patients about the risks and benefits of various RRT options prior to their initiation. There is also a growing interest in enhancing family involvement in RRT selection decisions, as family members often support and are affected by patients’ RRT decisions [15]. Educational resources describing the features, risks, and benefits of various medical treatment alternatives are increasingly employed to help patients and families make informed treatment choices [16-19] and could help patients with chronic kidney disease (CKD) better understand numerous considerations that could influence their choice of RRT. To date, however, little work has been done to identify the types of information patients and families might value most. It is also unclear whether African Americans’ and non-African Americans’ preferences for information might differ.

We performed qualitative structured group interviews among African American and non-African American patients with CKD and their families to elicit their views regarding information they felt should be featured in educational resources informing RRT selection decisions.

## Methods

### Rationale and study design

Our overall goal was to identify the types of information patients with CKD and families would view as important to include in educational resources informing others’ RRT selection decisions. We explored four a priori hypotheses in our study. First, we hypothesized that patients and families would view a broad variety of factors as important to include in educational resources. Second, we hypothesized that patients’ perceived informational needs would vary from those of family members, who would have less direct experience with RRTs and would experience RRT mostly in the role of caregivers. Third, in light of well-documented race differences in the types of RRTs initiated in the U.S [6,9,20], we hypothesized that patients’ and families’ informational needs might vary by race. Fourth, we hypothesized patients with and without prior RRT experience would have different views about the types of information that might best inform RRT selection decisions. For example, we hypothesized patients and families with advanced CKD (non-dialysis dependent) who had not previously experienced RRTs might be concerned about transitioning to ESRD. In contrast, we hypothesized patients and families who had previously experienced different RRT modalities might articulate experiences related to receiving these modalities but might not recall concerns they had prior to initiating RRT. We therefore gathered separate groups of patients with different RRT experiences (pre-ESRD, in-center hemodialysis, home hemodialysis, peritoneal dialysis, or transplant) and family members, and stratified groups by African American or non-African American self-reported race. This provided a total of 20 structured groups (5 African American and 5 non-African American patient groups, with one group per race for each treatment experience; 5 African American and 5 non-African American family member groups, with one group per race for each treatment experience).

### Study participants

We recruited study participants from community-based and academic nephrology practices affiliated with dialysis facilities as well as an academic kidney transplant center in the Baltimore, Maryland metropolitan area from September 2008 to July 2009. Participants were eligible for participation if they spoke English, were at least 18 years of age, had advanced, progressive CKD as determined by their nephrologists (described as “pre-ESRD”), had been on a RRT for at least a year prior to recruitment in the study (in-center hemodialysis, home hemodialysis, or peritoneal dialysis), or had received a kidney from a live donor (transplant). Nephrology practices and the transplant center provided us with lists of potentially eligible participants. We first recruited patients for participation, and then asked them to identify one family member or friend (referred to as “family member”) involved in their ESRD treatment decisions. All participants completed a written questionnaire describing their demographic characteristics and their relationship to patient participants (for family members or friends). The Johns Hopkins School of Medicine Institutional Review Board approved all protocols and consent procedures.

### Structured group interviews

We designed group interviews to obtain tabulated and open-ended feedback from participants regarding their views on the types of information they felt were most important to address in educational resources about selecting RRTs. Trained moderators used standard guides to conduct structured group interviews. Moderators provided each group participant with a written itemized list of 36 factors reported by patients in prior studies to influence their RRT experiences [3,5,6,21]. All patients and family members received the same list with the exception of items specific to a RRT modality. For example, patients and family members with kidney transplants received items related to transplantation (e.g., “finding a living donor” and “getting on the waiting list”) but not related to dialysis fistulas or catheters. We grouped factors into seven domains:

Domain 1: Morbidity/mortality

Living longer

Going to the hospital

Infections

Complications with surgery

Cancer (asked of Pre-ESRD and Transplant groups only)

Making frequent trips to the doctor

Domain 2: Autonomy

Having children or getting pregnant

Control over treatment schedule

Doing the things I want to do when I want to do them

Going places by oneself

What I can eat or drink

Freedom and control over my life

Washing, dressing, eating and going to the toilet by oneself

Doing usual activities

Domain 3: Treatment delivery

Blood tests, x-rays, and doctor visits

Pills that must be taken

Providing my own treatment

Surgery (asked of Pre-ESRD and Transplant groups only)

Ordering and storing supplies at home

Surgery for fistulas or catheters (asked of Hemodialysis and Peritoneal groups only)

Fistula or catheter problems (asked of Hemodialysis and Peritoneal groups only)

The treatment (dialysis/transplant) going the way expected

Getting on the waiting list (asked of Transplant groups only)

Finding a living donor (asked of Transplant groups only)

Domain 4: Symptoms

Itching, cramping, or aching

Thinking clearly

Feeling tired

Gaining weight

Losing weight

Pain

Domain 5: Relationships

Having and enjoying sexual relations

How much family and friends need to help

Strains in ties with my family and friends

Making new friends

Domain 6: Psychological

Feeling sad, anxious, or stressed out

Domain 7: Finances

Money spent from my own pocket

Group interviews progressed through three stages. In the first stage, moderators explained the purpose of the interview and posed open-ended questions to probe participants’ prior experiences with initiating RRT [22]. Moderators then provided each participant with the itemized list of 36 factors and said, “Here is a list of factors patients who have kidney disease have said are important aspects of their kidney disease treatments. Please spend the next few minutes choosing the 3 most important aspects of treatment you think other patients/families facing decisions about kidney disease treatments should consider.” When addressing pre-ESRD groups, the moderators slightly modified the latter half of the statement and said, “If you/your family member were going to choose a kidney disease treatment today, which 3 things would you consider most important to you as you decide/help your family member decide on one treatment to pursue?” Moderators then asked all participants to circle three factors (in no particular order) they felt were most important to address in educational resources about RRT. Once participants circled three factors, the discussion progressed to a second stage (referred to as round 1), in which moderators asked each group participant to reveal their selected factors and to discuss with others their rationale for selections. In the third stage (referred to as round 2), moderators asked participants to again circle three factors they felt should be presented in educational resources about RRT options, taking into account their feelings about the group discussion from stage two. We audio-recorded and transcribed all group discussions verbatim.

### Analysis

We tallied the factors participants selected as important during the third stage of discussions. We considered a factor to be important if at least one participant selected that factor within their respective group. We described factors selected by groups and noted similarities in factors selected across groups. In addition, three trained investigators independently reviewed study transcripts to identify quotes reflecting participants’ rationale surrounding commonly identified factors to provide a context for their selection.

## Results

### Participant characteristics

The 68 patient participants (37 African Americans, 31 non-African Americans) and 62 family members (32 African Americans, 30 non-African Americans) were demographically diverse. Most participants were non-Hispanic and had health insurance. Family members were comprised primarily of patients’ children, spouses and siblings (Tables 1 and 2).

**Table 1:** African American and non-African American patient characteristics

	**Pre-ESRD**		**Hemodialysis**		**Home hemodialysis**		**Peritoneal dialysis**		**Transplant**	
	**AA (n=6)**	**Non-AA (n=7)**	**AA (n=7)**	**Non- AA (n=8)**	**AA (n=4)**	**Non-AA (n=1)**	**AA (n=9)**	**Non-AA (n=4)**	**AA (n=11)**	**Non-AA (n=11)**
**Ethnicity**										
Hispanic	0 (0)	0 (0)	1 (14)	0 (0) ^¥^	0 (0)	0 (0)	0 (0)	1 (25)	0 (0) ^¥^	0 (0)^¥^
**Race**										
White	0 (0)	5 (71)	0 (0)	8 (100)	0 (0)	1 (100)	0 (0)	3 (75)^€^	0 (0)	11 (100)
Black	6 (100)	0 (0)	7 (100)	0 (0)	4 (100)	0 (0)	9 (100)	0 (0)^€^	11 (100)	0 (0)
Other	0 (0)	2 (29)	0 (0)	0 (0)	0 (0)	0 (0)	0 (0)	0 (0)^€^	0 (0)	0 (0)
**Age**										
Mean	54	58	56	65^£^	53	78	53	59	50	55^£^
[Range]	[28–69]	[24–82]	[27–65]	[55–80]	[38–75]	[N/A]	[33–69]	[53–74]	[37–61]	[18–65]
**Gender**										
Female	5 (83)	2 (29)	3 (43)	4 (50)	1 (25)	0 (0)	6 (66)	0 (0)	6 (65)	8 (73)
**Education**										
HS or less	3 (50)	0 (0)	0 (0)	4 (50)	1 (25)	0 (0)	2 (22)	3 (75)	4 (36)	2 (18)
At least two years of college	3 (50)	7 (100)	7 (100)	4 (50)	3 (75)	1 (100)	7 (77)	1 (25)	7 (64)	9 (82)
**Marital Status**										
Married/ living with partner	0 (0)	6 (86)	1 (14)	6 (76)	3 (75)	0 (0)	2 (22)	3 (75)	8 (73)	8 (73)
**Health Insurance**										
Insured	6 (100)	7 (100)	7 (100)	7 (87)^†^	4 (100)	1 (100)	9 (100)	4 (100)	11 (100)	11 (100)

*AA abbreviated for African American.

¥ Does not total to 100%; non-AA HD missing 3 (37), AA transplant missing 3 (28), non-AA transplant missing 2 (18).

€ Does not total to 100%; non-AA PD missing 1(25).

£Does not total to 100%; non-AA HD missing 1 (13), non-AA transplant missing 2 (18).

† Does not total to 100%; non-AA HD missing 2 (25).

**Table 2:** African American and non-African American family member characteristics

	**Pre-ESRD**		**Hemodialysis**		**Home hemodialysis**		**Peritoneal dialysis**		**Transplant**	
	**AA (n=6)**	**Non-AA (n=7)**	**AA (n=7)**	**Non-AA (n=6)**	**AA (n=3)**	**Non-AA (n=3)**	**AA (n=7)**	**Non-AA (n=3)**	**AA (n=9)**	**Non-AA (n=11)**
**Ethnicity**										
Hispanic	0 (0) ^¥^	0 (0)	0 (0)	1 (14)^¥^	0 (0)	1 (33)	0 (0)	1 (33)^¥^	0 (0)	2 (18)^¥^
**Race**										
White	0 (0)	6 (86)	0 (0)	6 (100)	0 (0)^*^	1 (33)^*^	0 (0)	2 (66)	0 (0)	10 (91)
Black	6 (100)	0 (0)	7 (100)	0 (0)	3 (100)^*^	0 (0)^*^	7 (100)	0 (0)	9 (100)	0 (0)
Other	0 (0)	1 (14)	0 (0)	0 (0)	0 (0)^*^	1 (33)^*^	0 (0)	1 (33)	0 (0)	1 (9)
**Age**										
Mean [Range]	47^€^	65^€^	55	62.5	45^€^	37^€^	45	56	55.5	60
	[29–63]	[55–78]	[46–67]	[44–80]	[37–52]	[N/A]	[45–75]	[50–62]	[39–68]	[23–79]
**Gender**										
Female	5 (83)	5 (71)	5 (71)	5 (83)	3 (100)	3 (100)	4 (57)	3 (100)	5 (55)	3 (27)
**Education**										
HS or less	3 (50)	2 (29)	5 (71)	4 (67)	0 (0)	0 (0)	2 (28)	2 (66)	4 (44)	3 (27)
At least 2 years of college	3 (50)	5 (71)	2 (28)	2 (33)	3 (100)	3 (100)	5 (71)	1 (33)	5 (56)	8 (73)
**Marital Status**										
Married/ living with partner	3 (50)	7 (100)	4 (57)	5 (83)	2 (67)	2 (67)	4 (57)	3 (100)	5 (56)	11(100)
**Health Insurance**										
Insured	5 (83)	7 (100)	5 (71)^†^	6 (100)	3 (100)	3 (100)	7 (100)	3 (100)	9 (100)	11(100)
**Relationship to Patient**										
Spouse	0 (0)	6 (86)	1 (14)	4 (67)	2 (67)	1 (33)	1 (14)	3 (100)	3 (33)	8 (73)
Parent/Parent-in-law	0 (0)	0 (0)	2 (28)	0 (0)	0 (0)	0 (0)	0 (0)	0 (0)	2 (22)	2 (18)
Child	2 (33)	1 (14)	1 (14)	1 (17)	0 (0)	0 (0)	0 (0)	0 (0)	1 (11)	0 (0)
Sibling	2 (33)	0 (0)	2 (28)	0 (0)	1 (33)	1 (33)	4 (57)	0 (0)	0 (0)	0 (0)
Cousin	0 (0)	0 (0)	0 (0)	0 (0)	0 (0)	0 (0)	0 (0)	0 (0)	2 (22)	0 (0)
Other/Friend	2 (33)	0 (0)	0 (0)	1 (17)	0 (0)	1 (33)	2 (28)	0 (0)	1 (11)	1 (9)

*AA abbreviated for African American; ¥ Does not total to 100%; AA pre-ESRD missing 1 (17), AA HD missing 1 (17), AA PD missing 1 (14), AA transplant missing 1 (11); ^*^Does not total to 100%, non-AA HHD missing 1 (33); € Does not total to 100%; AA pre-ESRD missing 1 (17), non-AA pre-ESRD missing 1 (14), AA HHD missing 1 (33), non-AA HD missing 2 (67); † Does not total to 100%; AA HD family missing 1 (14).

### Range of information most desired by African American and non-African American patients

Patients frequently selected factors pertaining to morbidity or mortality, autonomy, treatment delivery, and symptoms as important to address in educational resources about RRT selection decisions (Table 3). Patients only mentioned factors on the predefined lists provided to them and did not cite additional factors.

**Table 3:** Patients’ and family members’ factors to address in educational resources about RRT selection decisions

	Ranking round*	All patients n(%)**		All family members n(%)**	
		**AA**	**Non-AA**	**AA**	**Non- AA**
		**N=37**	**N=31**	**N=32**	**N=30**
**Morbidity/Mortality**					
Living Longer	1	30 (81)	22 (71)	22 (69)	23 (77)
	2	22 (59)	21 (68)	17 (53)	23 (77)
Going to the hospital	1	6 (16)	3 (10)	2 (6)	2 (7)
	2	2 (5)	2 (6)	1 (3)	0 (0)
Infections	1	2 (5)	5 (16)	10 (31)	6 (20)
	2	1 (3)	3 (10)	7 (22)	3 (10)
Complications with surgery	1	1 (3)	2 (6)	4 (13)	2 (7)
	2	0 (0)	2 (6)	3 (9)	1 (3)
Cancer	1	2 (5)	1 (3)	0 (0)	0 (0)
	2	0 (0)	2 (6)	0 (0)	0 (0)
Making frequent trips to the doctor	1	1 (3)	0 (0)	1 (3)	2 (7)
	2	5 (14)	1 (3)	2 (6)	1 (3)
**Autonomy**					
Having children or getting pregnant	1	0 (0)	0 (0)	2 (6)	2 (7)
	2	0 (0)	0 (0)	3 (9)	2 (7)
Control over treatment schedule	1	6 (16)	5 (16)	6 (19)	4 (13)
	2	2 (5)	5 (16)	2 (6)	3 (10)
Doing the things I want to do when I want to do them	1	10 (27)	11 (35)	10 (31)	7 (23)
	2	7 (19)	13 (42)	6 (19)	7 (23)
Going places by oneself	1	5 (14)	3 (10)	2 (6)	1 (3)
	2	1 (3)	2 (6)	0 (0)	1 (3)
What I can eat or drink	1	4 (11)	4 (13)	4 (13)	5 (17)
	2	4 (11)	4 (13)	5 (16)	6 (20)
Freedom and control over my life	1	14 (38)	8 (26)	10 (31)	14 (47)
	2	6 (16)	7 (23)	8 (25)	14 (47)
Washing, dressing, eating, and going to the toilet by myself	1	1 (3)	1 (3)	0 (0)	2 (7)
	2	0 (0)	1 (3)	1 (3)	2 (7)
Doing usual activities	1	3 (8)	3 (10)	2 (6)	5 (17)
	2	4 (11)	3 (10)	5 (16)	6 (20)
**Delivery**					
Blood tests, x-rays, and doctors visits	1	3 (8)	3 (10)	2 (6)	6 (20)
	2	0 (0)	3 (10)	0 (0)	6 (20)
Pills that must be taken	1	4 (11)	4 (11)	1 (3)	7 (23)
	2	3 (8)	4 (13)	1 (3)	8 (27)
Providing my own treatment	1	6 (16)	3 (10)	2 (6)	0 (0)
	2	3 (8)	1 (3)	1 (3)	1 (3)
Surgery	1	2 (5)	1 (3)	0 (0)	1 (3)
	2	0 (0)	1 (3)	0 (0)	0 (0)
Ordering and storing supplies at home	1	2 (5)	0 (0)	1 (3)	2 (7)
	2	3 (8)	0 (0)	1 (3)	1 (3)
Surgery for fistulas or catheters	1	1 (3)	1 (3)	1 (3)	1 (3)
	2	1 (3)	3 (10)	1 (3)	1 (3)
Fistula or catheter problems	1	3 (8)	1 (3)	0 (0)	0 (0)
	2	3 (8)	1 (3)	1 (3)	1 (3)
The treatment going as expected	1	1 (3)	2 (6)	2 (6)	3 (10)
	2	4 (11)	2 (6)	0 (0)	2 (7)
Getting on the waiting list	1	0 (0)	2 (6)	1 (3)	1 (3)
	2	0 (0)	2 (6)	0 (0)	0 (0)
Finding a living donor	1	3 (8)	3 (10)	4 (13)	7 (23)
	2	0 (0)	4 (13)	4 (13)	8 (27)
**Symptoms**					
Itching, cramping, or aching	1	2 (5)	1 (3)	1 (3)	0 (0)
	2	2 (5)	1 (3)	0 (0)	0 (0)
Thinking clearly	1	5 (14)	4 (13)	5 (16)	0 (0)
	2	2 (5)	7 (23)	2 (6)	0 (0)
Feeling tired	1	1 (3)	3 (10)	3 (9)	3 (10)
	2	5 (14)	2 (6)	3 (9)	3 (10)
Gaining weight	1	1 (3)	2 (6)	0 (0)	0 (0)
	2	1 (3)	5 (16)	1 (3)	0 (0)
Losing weight	1	0 (0)	1 (3)	0 (0)	0 (0)
	2	1 (3)	0 (0)	2 (6)	0 (0)
Pain	1	2 (5)	0 (0)	4 (13)	2 (7)
	2	1 (3)	0 (0)	4 (13)	6 (20)
**Relationships**					
Having and enjoying sexual relations	1	1 (3)	0 (0)	1 (3)	1 (3)
	2	0 (0)	0 (0)	5 (16)	2 (7)
How much family and friends need to help	1	8 (22)	1 (3)	4 (13)	0 (0)
	2	1 (3)	1 (3)	4 (13)	0 (0)
Strains in ties with my family and friends	1	1 (3)	1 (3)	0 (0)	0 (0)
	2	1 (3)	0 (0)	1 (3)	0 (0)
Making new friends	1	0 (0)	0 (0)	0 (0)	0 (0)
	2	2 (5)	0 (0)	1 (3)	0 (0)
**Psychological**					
Feeling sad, anxious, stressed out	1	1 (3)	2 (6)	3 (9)	1 (3)
	2	2 (5)	1 (3)	3 (9)	2 (7)
**Finance**					
Money spent from my own pocket	1	2 (5)	1 (3)	3 (9)	0 (0)
	2	1 (3)	1 (3)	2 (6)	1 (3)

*Participants rankings in round 1 constituted “Stage 2” of the mixed methods study; rankings in round 2 constituted “Stage 3” of the mixed methods study; **Percentage of total persons among groups provided with this option to consider.

#### Morbidity or mortality

All ten patient groups (5 African American, 5 non-African American) selected at least one factor pertaining to the effect of RRT on patients’ morbidity or mortality. Selections included “living longer” and “making frequent trips to the doctor” (Table 3). A peritoneal dialysis patient explained:

"“I think that’s something that everyone needs to know that even though you do have kidney disease, it doesn’t mean that it’s a lost cause; it doesn’t mean that there is no hope. You go on dialysis and hopefully you can get a transplant… you definitely can live longer” (non-African American patient, peritoneal dialysis)."

#### Autonomy

Similarly, all ten patient groups (5 African American, 5 non-African American) also selected at least one factor pertaining to the effect of RRT on patients’ autonomy. Selections included “doing things I want to do when I want to do them” and “freedom and control over my life” (Table 3). One participant noted:

"“I can’t work. I was a senior executive for a national company and 80% of my time was spent in other states, and I can’t do that anymore…you can’t be a top executive and only work three days a week” (non-African American patient, in-center hemodialysis)."

#### Treatment delivery

Nine patient groups (5 African American, 4 non-African American) selected at least one factor pertaining to patients’ experiences with initiating RRT treatment delivery. Selections included “finding a living donor” and “fistula or catheter problems” (Table 3). One patient participant commented:

"“When I first started, a lot of patients that I met didn’t understand what the differences between the fistula and the catheter was, why, what was the purpose of either, and I just think that’s something they need to educate us on more for when you are a new patient” (non-African American patient, peritoneal dialysis)."

#### Symptoms

Five patient groups (2 African American, 3 non-African American) selected at least one factor pertaining to the influence of RRT on symptoms patients might experience. Selections included “thinking clearly” and “itching, cramping, or aching” (Table 3). One patient participant explained:

"“There needs to be more awareness of what happens to a person leading up to kidney failure because if I had known about the intense itching and everything I was suffering from and why that was happening, I would have sought treatment sooner and I might not have almost died” (non-African American patient, peritoneal dialysis)."

#### Relationships

Three African American patient groups selected factors pertaining to the influence of RRT on personal relationships. Selections included “family and friends need to help” and “making new friends” (Table 3). None of the non-African American patient groups selected this factor. One participant stated:

"“Friendship comes because we are all in the same boat. We know that everybody needs somebody sometimes. And the good part about it is that we can rely and we can converse, nobody gets angry, nobody gets upset” (African American patient, in-center hemodialysis)."

### Range of information most desired by African American and non-African American family members

Similar to patients, both African American and non-African American family members most frequently selected factors pertaining to the effect of RRT on patients’ morbidity or mortality, autonomy, experiences with treatment delivery, and their symptoms as important to address in educational resources. Within domains, family members sometimes chose different factors than patients. Family members also more frequently discussed the influence of RRT selection on patients’ psychological well-being and finances (Table 3). Family members only identified factors on the predefined list provided to them and did not cite additional factors.

#### Morbidity or mortality

All ten family member groups (5 African American, 5 non-African American) selected at least one factor pertaining to the effect of RRT on patients’ morbidity or mortality. Selections included “living longer” and “infections” (Table 3). A peritoneal dialysis family member explained:

"“You have to know that dealing with the kidneys and the treatments and things that there are infections that you have to worry about. You need information on how you can handle it and move on” (African American family member, peritoneal dialysis)."

#### Autonomy

Eight family member groups (4 African American, 4 non-African American) chose at least one factor pertaining to the effect of RRTs on patients’ autonomy. Selections included “freedom and control over my life” and “what I can eat or drink” (Table 3).

One family member commented:

"“He wanted to continue to eat the way he had always eaten and I mean I sat day and night reading everything. When we went to the market I had my list to say this is what you can eat, this is what you can’t because he still wanted to eat lunch meats, anything that was high in salt, and you know we almost had a bad one” (African American family member, in-center hemodialysis)."

#### Treatment delivery

Four family member groups (2 African American, 2 non-African American) selected at least one factor pertaining to patients’ experiences with initiating RRT treatment delivery. Selections included “finding a living donor” and “ordering/storing supplies at home” (Table 3). One family member noted:

"“There’s a lot of boxes-there’s a lot of supplies that come in, all the medical stuff. If you have not prepared for all that’s involved in getting your dialysis treatment delivered into your home and the influx of materials that’s coming in it can be very cumbersome. I think it’s important that people are aware of the types of things that are going to come in and the full scope of what has to be done - you have to rearrange your home” (African American family member, peritoneal dialysis)."

#### Symptoms

Four family member groups (3 African American, 1 non-African American) chose at least one factor pertaining to the influence of RRT on symptoms patients might experience. Selections included “feeling tired” and “pain” (Table 3). One family member stated:

"“It’s something that I think is important to know about beforehand, what to expect, and thinking along the lines of either dialysis or transplant, whatever one, how much pain is involved and how much discomfort there is going to be” (African American family member, pre-ESRD)."

#### Relationships, psychological well-being, and finances

Three African American family member groups identified factors pertaining to patients’ relationships as important factors to address in educational resources about RRT selection. Selections included “family and friends need to help” and “having and enjoying sexual relations.” None of the non-African American family member groups selected these factors. Two groups (1 African American, 1 non-African American) also selected a factor pertaining to the psychological impact of RRT selection (i.e., “feeling sad, anxious or stressed out”) and one African American group (but no non-African American groups) chose a factor pertaining to the influence of RRT on personal finances (Table 3). One family member described the psychological impact her family member experienced while facing the need to make a decision about RRT initiation:

"“She feels that dialysis is the end of anybody’s life. Once you go on dialysis, that is it, there is nothing left. She knows so many people that had it and then they die, so she is fearful and angry about that” (African American family member, pre-ESRD)."

### Evaluation of differences in information needs by race and experience with ESRD treatments

We did not detect substantial differences in participants’ reported information needs according to their race or ethnicity (Table 3). In analyses exploring differences in information needs among patients with varying experiences on ESRD therapies, patients with no prior experience reported they felt it important to have educational resources address a broader range of factors compared to patients with experience on various treatments for ESRD (Additional file 1: Appendix Tables 1 and 2).

### Effect of discussions on participants’ perceived information needs

Discussion among participants between rounds 1 and 2 of the ranking exercise did not appear to substantially change their perceived information needs. However, participants did seem to shift their ranking somewhat after round 1 discussions. For example, more patients and family members ranked “living longer” as important to feature in educational resources in the first round than in the second round (Table 3).

## Discussion

Educational resources addressing factors that patients and families deem most important to their decisions regarding RRT could facilitate a RRT selection well-aligned with their personal values. In this study, patients with CKD and their families most frequently viewed information on how RRTs affect patients’ morbidity or mortality, autonomy, experiences with treatment delivery, symptoms, personal relationships, psychological well-being, and finances as important factors to address in educational resources informing RRT selection decisions. Family members additionally identified information on how RRTs affect patients’ psychological well-being and finances. Most findings were similar between African American and non-African American patients and families. However, African American patients and families more frequently identified the influence of RRTs on personal relationships and finances as important factors to address compared to non-African Americans.

To our knowledge, this is the first US study to explore ethnic/race differences in the types of information patients and their families feel should be included in educational resources to support RRT decisions. While prior studies have informed the development of educational resources for patients related to CKD care [23] and the transition to traditional dialysis therapies (i.e., hemodialysis and peritoneal dialysis) [24], they have not specifically focused on identifying key information that would help patients and families understand critical differences between a range of RRT modalities, including home hemodialysis or transplantation [3,25]. They have not also sought to identify factors that might enhance the cultural relevance of materials for African Americans or other minority groups.

Our findings suggest that family members may broaden the range of considerations influencing RRT decisions beyond considerations commonly expressed by patients. Family members may provide both psychological and cognitive support during the decision-making process and are also likely to play significant caregiver roles (e.g., providing transportation to dialysis and medical appointments). Efforts to include information about factors deemed important to family members could not only help families better understand the health risks and benefits of various RRTs but may also help them set reasonable expectations regarding the logistical and psychological burden certain RRT choices may place on patients’ families.

Our findings also highlight the potential importance of tailoring educational resources to meet the needs of patients and their families based on their prior experiences with ESRD treatments. For instance, we found that patients with no experience on ESRD treatments expressed interest in a broader range of topics when compared to their counterparts who had prior experience with therapies. Patients with no prior experience with ESRD treatments may need extensive education on how ESRD and its treatments could impact multiple aspects of their lives, while patients with treatment experience may need more focused information about how treatment alternatives might change their treatment experiences. Notably, our participants’ group discussions may have influenced their final rankings of factors they felt should be important to include in educational materials. For instance, more participants rated “living longer” as important to discuss in educational materials during the first round of ranking compared to the second round of ranking. It is possible discussion of the importance of various factors pertaining to treatment experience may have encouraged our participants to consider more patient-centered aspects of treatment (e.g., such as the influence of treatments on patients’ relationships) that could influence others’ treatment choices.

Limitations of our study deserve mention. First, the experiences of patients recruited in Baltimore, Maryland may not generalize to the experiences of other patients with ESRD and their families, or individuals from different geographic areas. Because we recruited patients receiving RRT for more than a year, it is probable that patients’ recall concerning decision making about RRT initiation could have changed over time. It is also likely patients’ recollections regarding initiation of a single RRT could have been altered since there were some participants who had experience with more than one treatment modality. Second, some of our patient and family groups were small and may not have elicited the full range of factors considered important to more representative groups of patients and families. Many contextual factors could influence patients’ and their family members’ perceived information needs, including their education levels, financial resources, available family support, and independence. Family members’ perceived information needs might also vary according to the closeness of their relationships with patients. Moreover, the total number of participants in our groups was small, limiting our ability to make inferences regarding whether participants’ information needs might vary according to these factors. Our findings of potential differences between African American and non-African American groups could be influenced by differences in the education or financial status of participants and should therefore be interpreted with caution. Finally, we asked patients to identify one family member to participate in our study. The perspectives of the family members chosen to participate may have differed from the viewpoints of non-participants. Notwithstanding these limitations, we are unaware of other studies designed to simultaneously identify patients’ and families’ needs regarding education to support RRT selection decisions. Furthermore, we are not aware of studies exploring potential race differences in these needs.

## Conclusions

African American and non-African American patients with CKD and their families reported that educational resources informing RRT decisions should include information on how RRTs could affect patients’ morbidity or mortality, autonomy, experiences with RRT delivery, symptoms, psychological well-being, personal relationships and finances. Educational resources addressing these factors could help ensure patients’ and families’ RRT selection decisions are well-aligned with their personal values.

## Abbreviations

RRT: Renal replacement therapy; ESRD: End-stage renal disease; LKT: Live kidney transplantation.

## Competing interests

Support came from Grant #R01DK079682 from the National Institute of Diabetes and Digestive and Kidney Diseases (Drs. Boulware; Rabb; Powe), Grant #K23DK070757 from the National Center for Minority Health and Health Disparities and the National Institute of Diabetes and Digestive and Kidney Diseases (Dr. Boulware), Grant from the Harold Amos Faculty Development Program of the Robert Wood Johnson Foundation(Dr. Crews), Grant # 5KL2RR025006 from the National Center for Research Resources (NCRR), a component of the National Institutes of Health (NIH) and the NIH Roadmap for Medical Research (Dr. Greer), and Grant # 3R01DK079682-03S1 (Dr. Greer).

The sponsor had no role in the study design; collection, analysis, or interpretation of the data. The sponsor also did not participate in writing the report or the decisions to submit the report for publication.

The authors declare that they have no competing interests.

## Authors’ contributions

ND made substantial contributions to analysis and interpretation of data, and was involved in drafting the manuscript and revising it critically for important intellectual content. PLE made substantial contributions to conception and design, acquisition of data, analysis and interpretation of data, and drafting and revising the manuscript. JA made substantial contributions to analysis and interpretation of data, and drafting the manuscript and revising it critically for important intellectual content. LLB made substantial contributions to acquisition of data, analysis and interpretation of data, and was involved in drafting the manuscript and revising it critically for important intellectual content. DCC made contributions to drafting the manuscript and revising it critically for important intellectual content. RCG made contributions to drafting the manuscript and revising it critically for important intellectual content. HR contributed substantially to the study conception and design, analysis and interpretation of data, and drafting the manuscript and revising it critically for important intellectual content. NRP contributed substantially to the study conception and design, analysis and interpretation of data, and drafting the manuscript and revising it critically for important intellectual content. BGJ made substantial contributions to acquisition of data, analysis and interpretation of data, and drafting the manuscript and revising it critically for important intellectual content. LG made substantial contributions to acquisition of data, analysis and interpretation of data, and drafting the manuscript and revising it critically for important intellectual content. PA made substantial contributions to analysis and interpretation of data, and was involved in drafting the manuscript and revising it critically for important intellectual content. MJ made substantial contributions to acquisition of data, analysis and interpretation of data, and was involved in drafting the manuscript and revising it critically for important intellectual content. LEB made substantial contributions to conception and design, acquisition of data, analysis and interpretation of data, and drafting the manuscript and revising it critically for important intellectual content. All authors read and approved the final manuscript.

## Pre-publication history

The pre-publication history for this paper can be accessed here:

http://www.biomedcentral.com/1471-2369/14/9/prepub

## Supplementary Material

Additional file 1 **Appendix Table 1.** Patients’ factors to address in educational resources about RRT selection decisions. **Table 2.** Family members’ factors to address in educational resources about RRT selection decisions. Click here for file
