# Supplementary material for: Selecting renal replacement therapies: what do African American and non-African American patients and their families think others should know? A mixed methods study
Source: BMC Nephrol. 2013 Jan 14;14:9. doi: 10.1186/1471-2369-14-9 (PMC3565884; doi:10.1186/1471-2369-14-9)
Supplement: Additional file 1 — Appendix Table 1. Patients’ factors to address in educational resources about RRT selection decisions. Table 2. Family members’ factors to address in educational resources about RRT selection decisions. [file 1471-2369-14-9-S1.docx]

Table 1. Patients’ factors to address in educational resources about RRT selection decisions

|  | **Ranking Round*** | **Pre ESRD n (%)** | | **HD n (%)** | | **HHD n (%)** | | **PD n (%)** | | **TR n (%)** | |
| --- | --- | --- | --- | --- | --- | --- | --- | --- | --- | --- | --- |
|  |  | **AA** | **Non- AA** | **AA** | **Non- AA** | **AA** | **Non- AA** | **AA** | **Non- AA** | **AA** | **Non- AA** |
|  |  | **N=6** | **N=7** | **N=7** | **N=8** | **N=4** | **N=1** | **N=9** | **N=4** | **N=11** | **N=11** |
| **Morbidity/Mortality** | | | | | | | | | | | |
| Living Longer | 1 | 3(50) | 7(100) | 7(100) | 5(63) | 4(100) | 1 (100) | 6(67) | 2(50) | 10(91) | 7(64) |
|  | 2 |  | 5(71) | 3(43) | 6(75) | 4(100) | 1 (100) | 6(67) | 3(75) | 9 (82) | 6 (55) |
| Going to the hospital | 1 | 1(17) | 1 (14) | 5(71) | 2(25) |  |  |  |  |  |  |
|  | 2 |  |  | 2(29) | 2(25) |  |  |  |  |  |  |
| Infections | 1 | 1(17) | 3(43) | 1(14) |  |  |  |  | 2(50) |  |  |
|  | 2 |  | 2(29) | 1(14) |  |  |  |  | 1(25) |  |  |
| Complications with surgery | 1 | 1(17) | 2(29) |  |  |  |  |  |  |  |  |
|  | 2 |  | 2(29) |  |  |  |  |  |  |  |  |
| Cancer | 1 | 2(33) | 1(14) | --- | -- | -- | -- | -- | -- |  |  |
|  | 2 |  | 2(29) | --- | -- | -- | -- | -- | -- |  |  |
| Making frequent trips to the doctor | 1 |  |  |  |  |  |  | 1(11) |  |  |  |
|  | 2 | 2(33) |  | 2(29) |  |  |  | 1(11) | 1(25) |  |  |
| **Autonomy** | | | | | | | | | | | |
| Having children or getting pregnant | 1 |  |  |  |  |  |  |  |  |  |  |
|  | 2 |  |  |  |  |  |  |  |  |  |  |
| Control over treatment schedule | 1 |  |  |  |  |  |  | 3(33) | 1(25) | 3(27) | 4(36) |
|  | 2 | 1(17) | 1(14) |  | 1(13) |  |  | 1(11) |  |  | 3(27) |
| Doing the things I want to do when I want to do them | 1 | 4(67) | 2(29) | 2(29) | 2(25) | 2(50) | 1 (100) | 1(11) | 2(50) | 1(9) | 4(36) |
|  | 2 | 3(50) | 4(57) | 3(43) | 2(25) | 1(25) | 1 (100) |  | 2(50) |  | 4(36) |
| Going places by oneself | 1 | 2(33) | 1(14) | 2(29) | 2(25) |  |  | 1(11) |  |  |  |
|  | 2 | 1(17) |  |  | 1(13) |  |  |  | 1(25) |  |  |
| What I can eat or drink | 1 | 2(33) | 1(14) | 1(14) | 2(25) |  |  | 1(11) |  |  | 1(9) |
|  | 2 | 2(33) | 1(14) | 1(14) | 2(25) |  |  | 1(11) |  |  | 1(9) |
| Freedom and control over my life | 1 | 2(33) | 2(29) | 2(29) | 3(38) | 1(25) |  | 5(56) |  | 4(36) | 3(27) |
|  | 2 | 1(17) | 2(29) |  | 3(38) |  |  | 5(56) |  |  | 2(18) |
| **Autonomy Continued** | | | | | | | | | | | |
| Washing, dressing, eating, and going to the toilet by myself | 1 | 1(17) | 1(14) |  |  |  |  |  |  |  |  |
|  | 2 |  | 1(14) |  |  |  |  |  |  |  |  |
| Doing usual activities | 1 | 1(17) |  | 2(29) | 1(13) |  |  |  | 1(25) |  | 1(9) |
|  | 2 | 1(17) |  | 2(29) | 2(25) |  |  | 1(11) |  |  | 1(9) |
| **Delivery** | | | | | | | | | | | |
| Blood tests, x-rays, and doctors visits | 1 | 2(33) | 1(14) |  | 1(13) |  |  |  |  | 1(9) | 1(9) |
|  | 2 |  | 1(14) |  | 1(13) |  |  |  |  |  | 1(9) |
| Pills that must be taken | 1 |  | 1(14) | 2(29) |  |  |  |  |  | 2(18) | 3(27) |
|  | 2 | 3(50) | 1(14) |  |  |  |  |  |  |  | 3(27) |
| Providing my own treatment | 1 | 1(17) | 2(29) |  |  | 2(50) | 1 (100) | 3(33) |  |  |  |
|  | 2 | 1(17) |  |  |  |  |  | 2(18) | 1(25) |  |  |
| Surgery | 1 | 2(33) | 1(14) | --- | --- | --- | --- | --- | --- |  |  |
|  | 2 |  | 1(14) | --- | --- | --- | --- | --- | --- |  |  |
| Ordering and storing supplies at home | 1 | 1(17) |  |  |  |  |  | 1(11) |  |  |  |
|  | 2 |  |  |  |  |  |  | 3(33) |  |  |  |
| Surgery for fistulas or catheters | 1 | --- | --- |  | 1(13) |  |  | 1(11) |  | --- | --- |
|  | 2 | --- | --- |  | 2(25) |  |  | 1(11) | 1(25) | --- | --- |
| Fistula or catheter problems | 1 | --- | --- | 2(29) | 1(13) | 1(25) |  |  |  | --- | --- |
|  | 2 | --- | --- | 2(29) |  |  | 1(100) | 1(11) |  | --- | --- |
| The treatment going as expected | 1 |  | 1(14) |  |  |  |  | 1(11) | 1(25) |  |  |
|  | 2 | 1(17) | 1(14) | 2(29) |  |  |  | 1(11) | 1(25) |  |  |
| Getting on the waiting list | 1 |  |  |  |  |  |  |  |  |  | 2(18) |
|  | 2 |  |  |  |  |  |  |  |  |  | 2(18) |
| Finding a living donor | 1 | --- | --- | --- | --- | --- | --- | --- | --- | 3(27) | 3(27) |
|  | 2 | --- | --- | --- | --- | --- | --- | --- | --- |  | 4(36) |
| **Symptoms** | | | | | | | | | | | |
| Itching, cramping, or aching | 1 |  |  |  |  | 1(25) |  | 1(11) | 1(25) |  |  |
|  | 2 | 1(17) |  |  |  |  |  | 1(11) | 1(25) |  |  |
| Thinking clearly | 1 | 3(50) | 3(43) |  | 1(13) | 1(25) |  |  |  | 1(9) |  |
|  | 2 | 2(33) | 6(86) |  | 1(13) |  |  |  |  |  |  |
| Feeling tired | 1 | 1(17) | 1(14) |  |  |  |  |  |  |  | 2(18) |
|  | 2 | 5(83) | 1(14) |  |  |  |  |  |  |  | 1(9) |
| Gaining weight | 1 |  |  | 1(14) | 1(13) |  |  |  |  |  | 1(9) |
|  | 2 | 1(17) |  |  | 1(13) |  |  |  |  |  | 4(36) |
| Losing weight | 1 |  |  |  |  |  |  |  | 1(25) |  |  |
|  | 2 | 1(17) |  |  |  |  |  |  |  |  |  |
| Pain | 1 | 1(17) |  |  |  |  |  |  |  | 1(11) |  |
|  | 2 | 1(17) |  |  |  |  |  |  |  |  |  |
| **Relationship** | | | | | | | | | | | |
| Having and enjoying sexual relations | 1 |  |  |  |  | 1(25) |  |  |  |  |  |
|  | 2 |  |  |  |  |  |  |  |  |  |  |
| How much family and friends need to help | 1 |  | 1(14) |  |  |  |  | 1(11) |  | 7(64) |  |
|  | 2 |  | 1(14) |  |  |  |  | 1(11) |  |  |  |
| Strains in ties with my family and friends | 1 |  | 1(14) | 1(14) |  |  |  |  |  |  |  |
|  | 2 | 1(17) |  |  |  |  |  |  |  |  |  |
| Making new friends | 1 |  |  |  |  |  |  |  |  |  |  |
|  | 2 |  |  | 2(29) |  |  |  |  |  |  |  |
| **Psychological** | | | | | | | | | | | |
| Feeling sad, anxious, stressed out | 1 |  | 1(14) |  |  |  |  | 1(11) |  |  | 1(9) |
|  | 2 | 1(17) |  |  |  |  |  | 1(11) |  |  | 1(9) |
| **Finance** | | | | | | | | | | | |
| Money spent from my own pocket | 1 | 2(33) | 1(14) |  |  |  |  |  |  |  |  |
|  | 2 | 1(17) | 1(14) |  |  |  |  |  |  |  |  |

*Participants rankings in round 1 constituted “Stage 2” of the mixed methods study; rankings in round 2 constituted “Stage 3;” **Percentage of total persons among groups provided with this option to consider

Table 2. Family members’ factors to address in educational resources about RRT selection decisions

|  | **Ranking Round*** | **Pre ESRD n (%)** | | **HD n (%)** | | **HHD n (%)** | | **PD n (%)** | | **TR n (%)** | |
| --- | --- | --- | --- | --- | --- | --- | --- | --- | --- | --- | --- |
|  |  | **AA** | **Non- AA** | **AA** | **Non- AA** | **AA** | **Non- AA** | **AA** | **Non- AA** | **AA** | **Non- AA** |
|  |  | **N=6** | **N=7** | **N=7** | **N=6** | **N=3** | **N=3** | **N=7** | **N=3** | **N=9** | **N=11** |
| **Morbidity/Mortality** | | | | | | | | | | | |
| Living Longer | 1 | 5(83) | 6(86) | 3(43) | 5(83) | 1(33) | 2(67) | 5(71) | 3(100) | 8(89) | 7(64) |
|  | 2 | 3(50) | 7(100) | 4(57) | 5(83) | 2(67) | 2(67) | 3(43) | 2(67) | 5(56) | 7(64) |
| Going to the hospital | 1 |  | 2(29) | 2(29) |  |  |  |  |  |  |  |
|  | 2 |  |  | 1(14) |  |  |  |  |  |  |  |
| Infections | 1 | 2(33) | 3(43) | 3(43) | 1(17) |  | 1(33) | 3(43) | 1(33) | 2(22) |  |
|  | 2 | 3(50) | 2(29) |  | 1(17) |  |  | 1(14) |  | 3(33) |  |
| Complications with surgery | 1 | 1(17) | 2(29) | 2(29) |  |  |  | 1(14) |  |  |  |
|  | 2 | 2(33) | 1(14) |  |  |  |  | 1(14) |  |  |  |
| Cancer | 1 |  |  | --- | --- | --- | --- | --- | --- |  |  |
|  | 2 |  |  | --- | --- | --- | --- | --- | --- |  |  |
| Making frequent trips to the doctor | 1 | 1(17) |  |  |  |  |  |  |  |  | 2(18) |
|  | 2 | 1(17) |  |  |  |  |  |  |  | 1(11) | 1(9) |
| **Autonomy** | | | | | | | | | | | |
| Having children or getting pregnant | 1 | 1(17) | 1(14) |  |  |  |  |  |  | 1(11) | 1(9) |
|  | 2 | 2(33) | 1(14) | 1(14) |  |  |  |  |  |  | 1(9) |
| Control over treatment schedule | 1 | 2(33) |  |  |  | 3(100) | 1(33) | 1(14) | 3(100) |  |  |
|  | 2 | 1(17) |  |  |  | 1(33) | 1(33) |  | 2(67) |  |  |
| Doing the things I want to do when I want to do them | 1 | 1(17) | 3(43) |  | 2(33) | 2 (67) | 1(33) | 3(43) | 1(33) | 4(44) |  |
|  | 2 | 1(17) | 3(43) |  | 2(33) |  |  | 3(43) | 1(33) | 2(22) | 1(9) |
| Going places by oneself | 1 |  | 1(14) | 1(14) |  |  |  |  |  | 1(11) |  |
|  | 2 |  |  |  |  |  |  |  | 1(33) |  |  |
| What I can eat or drink | 1 | 2(33) | 2(29) | 2(29) | 2(33) |  |  |  | 1(33) |  |  |
|  | 2 | 2(33) | 3(43) | 3(43) | 2(33) |  |  |  | 1(33) |  |  |
| Freedom and control over my life | 1 | 2(33) | 4(57) |  | 4(67) | 3(100) | 2(67) | 3(43) | 1(33) | 2(22) | 3(33) |
|  | 2 | 2(33) | 5(71) | 2(29) | 4(67) | 1(33) | 2(67) | 1(14) |  | 2(22) | 3(33) |
| Washing, dressing, eating, and going to the toilet by myself | 1 |  | 1(14) |  | 1(17) |  |  |  |  |  |  |
|  | 2 |  | 1(14) |  | 1(17) |  |  |  |  | 1(11) |  |
| Doing usual activities | 1 |  | 1(14) | 1(14) | 2(33) |  | 1(33) |  |  | 1(11) | 1(9) |
|  | 2 | 1(17) | 1(14) | 3(43) | 2(33) |  | 1(33) |  | 1(33) | 1(11) | 1(9) |
| **Delivery** | | | | | | | | | | | |
| Blood tests, x-rays, and doctors visits | 1 |  |  |  | 1(17) |  |  | 1(14) | 2(67) | 1(11) | 3(27) |
|  | 2 |  |  |  | 1(17) |  |  |  | 2(67) |  | 3(27) |
| Pills that must be taken | 1 | 1(17) | 1(14) |  |  |  |  |  |  |  | 6(67) |
|  | 2 |  | 2(29) | 1(14) |  |  |  |  | 1(33) |  | 5(45) |
| Providing my own treatment | 1 |  |  |  |  |  |  | 2(29) |  |  |  |
|  | 2 |  |  |  |  |  |  | 1(14) | 1(33) |  |  |
| Surgery | 1 |  | 1(14) |  |  |  |  |  |  |  |  |
|  | 2 |  |  |  |  |  |  |  |  |  |  |
| Ordering and storing supplies at home | 1 |  |  |  |  |  |  | 1(14) | 2(67) |  |  |
|  | 2 |  |  |  |  |  |  | 1(14) | 1(33) |  |  |
| Surgery for fistulas or catheters | 1 | --- | --- |  |  |  |  | 1(14) | 1(33) | --- | --- |
|  | 2 | --- | --- |  |  |  |  | 1(14) | 1(33) | --- | --- |
| Fistula or catheter problems | 1 | --- | --- |  |  |  |  |  |  | --- | --- |
|  | 2 | --- | --- |  |  |  |  | 1(7) | 1(33) | --- | --- |
| The treatment going as expected | 1 | 1(17) | 2(29) |  |  |  |  |  | 1(33) | 1(11) |  |
|  | 2 |  | 2(29) |  |  |  |  |  |  |  |  |
| Getting on the waiting list | 1 |  |  |  |  |  |  |  |  | 1(11) | 1(9) |
|  | 2 |  |  |  |  |  |  |  |  |  |  |
| Finding a living donor | 1 | --- | --- | --- | --- | --- | --- | --- | --- | 4(44) | 7(78) |
|  | 2 | --- | --- | --- | --- | --- | --- | --- | --- | 4(44) | 8(73) |
| **Symptoms** | | | | | | | | | | | |
| Thinking clearly | 1 | 1(17) |  | 2(29) |  |  |  | 1(14) |  | 1(11) |  |
|  | 2 |  |  |  |  |  |  | 1(14) |  | 1(11) |  |
| Itching, cramping, or aching | 1 |  |  | 1(14) |  |  |  |  |  |  |  |
|  | 2 |  |  |  |  |  |  |  |  |  |  |
| Feeling tired | 1 | 1(17) | 3(43) | 2(29) |  |  |  |  |  |  |  |
|  | 2 |  | 3(43) | 2(29) |  |  |  |  |  | 1(11) |  |
| Gaining weight | 1 |  |  |  |  |  |  |  |  |  |  |
|  | 2 |  |  |  |  |  |  |  |  | 1(11) |  |
| Losing weight | 1 |  |  |  |  |  |  |  |  |  |  |
|  | 2 | 1(17) |  | 1(14) |  |  |  |  |  |  |  |
| Pain | 1 | 4(67) | 1(14) |  |  |  | 1(33) |  |  |  |  |
|  | 2 | 2(33) | 1(14) | 1(14) |  |  |  |  |  | 1(11) | 5(45) |
| **Relationship** | | | | | | | | | | | |
| Having and enjoying sexual relations | 1 |  | 1(14) |  |  |  |  | 1(14) |  |  |  |
|  | 2 | 1(17) | 2(29) | 2(29) |  |  |  | 1(14) |  | 1(11) |  |
| How much family and friends need to help | 1 | 3(50) |  |  |  |  |  |  |  | 1(11) |  |
|  | 2 | 4(67) |  |  |  |  |  |  |  |  |  |
| Strains in ties with my family and friends | 1 |  |  |  |  |  |  |  |  |  |  |
|  | 2 | 1(17) |  |  |  |  |  |  |  |  |  |
| Making new friends | 1 |  |  |  |  |  |  |  |  |  |  |
|  | 2 |  |  |  |  |  |  |  |  | 1(11) |  |
| **Psychological** | | | | | | | | | | | |
| Feeling sad, anxious, or stressed out | 1 | 1(17) |  | 2(29) |  |  |  |  |  |  | 1(9) |
|  | 2 | 2(33) |  |  |  |  |  |  | 1(33) | 1(11) | 1(9) |
| **Finance** | | | | | | | | | | | |
| Money spent from my own pocket | 1 | 1(33) |  |  |  |  |  | 2(29) |  |  |  |
|  | 2 | 1(17) |  |  |  |  |  | 1(14) |  |  | 1(9) |

*Participants rankings in round 1 constituted “Stage 2” of the mixed methods study; rankings in round 2 constituted “Stage 3;” **Percentage of total persons among groups provided with this option to consider
